# Going Native. Emotion Science in the Twenty-First Century

**DOI:** 10.3389/fpsyg.2017.01212

**Published:** 2017-07-27

**Authors:** Beatrice de Gelder

**Affiliations:** ^1^Brain and Emotion Lab, Department of Cognitive Neuroscience, Faculty of Psychology and Neuroscience, Maastricht University Maastricht, Netherlands; ^2^Department of Computer Science, University College London London, United Kingdom; ^3^Italian Academy Institute of Advanced Studies, Columbia University New York, NY, United States

**Keywords:** emotion, consciousness, introspection, evolution, v, virtual reality

Emotion Science stuns the casual observer by the breadth of the issues that have been addressed in its long history; one need only look at the variety of chapters in the recent *Handbook of Emotion Science* (Barrett et al., 2016) to be impressed. Emotion research, or affective neuroscience as the recent name goes, ranges from animal and human to machine emotions and was recently extended to include robots and avatars. Its methods encompass neurobiological investigations, mainstream psychophysics experiments, studies on emotion regulation and clinical studies as well as extensive use of autobiographical and qualitative methods. Yet, since its beginnings the same persistent dichotomies concerning the nature of emotions continue to prevail. Theoretical positions have been defined again and again around conceptual contrasts that pitch emotional vs. rational or cognitive processes, basic vs. complex or constructed emotions, feelings vs. affect or emotions; implicit vs. explicit affective processes; automatic perception vs. to conscious experience or independent of it; emotion processes or emotional experience; emotions related or not to appraisal and cognition; personal vs. sub-personal processes; body vs. brain vs. mental processes; and arousal processes vs. appraisal processes.

There is no doubt that emotion science will gain in importance as scientific progress shifts the interest of researchers beyond understanding the sensory and cognitive endowment of organisms for passive perception to why and how the organism is motivated to act. In order to launch emotion science in the twenty-first century we need go beyond the classical debates that have shown surprising vitality to this day. Indeed, looking at the current dichotomies, including new proposals for transcending them, one wonders whether they may simply be novel ways of sidestepping the same old issues. That brings us to the question addressed here, of whether the time has come for a more radical turn.

Certainly this is not the first call for a radical move! Limiting ourselves to current debates, we see that researchers today are acutely aware of the many conceptual complexities and confusions and the need for an innovative stance to transcend the current dichotomies. Damasio (1999) has gone the furthest in conceptualizing emotions as patterns of bodily changes that combine somatic, visceral and musculoskeletal activities. Through ascending loops from the proto-self to awareness and through different levels of re-description and integration of organismic processes, the subjective emotional experience ultimately emerges and is rooted in everything before it. In contrast, Adolphs (2016) is in favor of maintaining a clear distinction between three different strata that together define what emotions are: the emotion states of the organism, our emotion concepts rooted in our language and culture and subjective emotional experiences. The notion of *function* is put in charge of bridging these strata and thereby building the inter-level connections or at least guaranteeing that these bridges will one day exist, even if few details are currently understood. The concept of functions plays many roles and to address this matter is well beyond our goal (see Fodor, 1968; Griffits, 1997). We only draw attention to two specific roles that the concept of function is expected to play here. One is emotions as performing a linguistic function defined by its semantic role, its role in the discourse on emotions in subjective experience and common sense guided introspection, which is what qualitative research methods most often build upon. The other meaning of function is that of a bridging principle or a set of translation rules. The assumption, or better, the hypothesis is that emotion scientists search for the functional correlates of the central concepts used to describe the affective information and explain emotion induced behavior related to it.

This position reflects the current consensus on how to anchor the familiar emotion concepts to the sciences, be it psychology, affective neuroscience or neurobiology, by viewing the characteristic emotion terms encountered at each of those levels as shorthand for functional roles that the basic organism' states play in behavior. To use the simplest example, typically, the emotion term *fear* is the name for the function of survival. The upshot is that the survival system is considered to be the fear system minus the subjective experience. (LeDoux, 2015) accepts the ensuing dichotomy between the functional description of emotion processes (i.e., survival), mostly implemented in the subcortical mechanisms and the relatively independent cortically-based processes (related to fear) that gives raise to feelings and emotion experience in humans. So the functional states denote the rock-bottom physical reality of the emotions and as the organismic roots of subjective affective experience.

This is not the place to detail the powerful objections to the notion of emotion primitives found in Griffits (1997) or the philosophical analyses and arguments against higher order theories by e.g., Carruthers (2016). Within the realm of the current emotion theory debates, higher order theories are embraced by some researchers such as Ledoux and rejected by others. For example, Barrett (2017) argues that assuming basic states (survival) with a functional translation that links them to subjective experience (fear), simply continues the mistake of assuming that there is a scientific basis to basic emotion states. Instead, she argues that emotions are cognitive constructs, fully masterminded for us by the linguistic and conceptual apparatus of society and culture. The subjective states we experience (fear) are not lawfully related to the organism (survival), the former are not caused by the latter, nor are they the functional correlates of them. Furthermore, they are not higher order descriptions of them because there is no rock-bottom, fact-of-the-matter level of emotions. Exit basic emotions, functionalism and higher order theories.

## Design functionalism as an empirical hypothesis

In the discussions we referred to so far, functionalism is viewed as perspective on how the different levels of emotion processes may be related to each other. Yet, there is also a very different meaning of functionalism at stake in biology and the life sciences. At its simplest, functionalism is the methodology that starts from behavioral analysis, focuses on design and isolates principles and searches for the mechanisms that generate them. Dennett (2017) has long championed this approach, calling it *reverse engineering*. It is a bottom up approach to understanding biological mechanisms, proceeding as an inductive discovery of design properties. It contrasts with the familiar approaches in emotion research that define a set of questions in a top down way, guided by a conceptual understanding (of what emotions are, which are basic, and what action tendencies are essential) and looking for the mechanisms of their implementation.

Dennett has forcefully argued that a major mistake in the sciences related to human behavior is the assumption that the explanations we give for a given behavior are only correct when they correspond to what an agent himself would provide when prompted. One could name it the “man in the mirror fallacy.” The fallacy consists in assuming that in studying behavior, the scientist is bound by his basic beliefs about how human actions ought to be explained. Dennett refers to this study of behavior and design as adopting the principle of “competence without comprehension.” Indeed, this has been the fallacy so far in emotion research: we attribute anger, fear or disgust when we assume that the agent's behavior is caused by one of these emotions. However, if, from a biological perspective, emotions are shorthand for descriptions of mechanisms of adaptive behavior, then there is no basis for such an assumption. A reaction of freezing may be eminently rational without any reference to fear experience or to any reasoning process in the organism. To define freezing as caused by fear does not advance our understanding of the mechanisms of the freezing reaction. Neither the subjective, not the linguistic, social or cultural analysis of fear brings us closer to understanding that mechanism.

How then is this functional approach to emotions to be implemented and how is it different from the basic emotion, the constructivist or any hybrid approach? The central proposal is that we need to return to the study of behavior. The way to do that is by endorsing new methodologies that allow a naturalistic study of behavior and to combine this with modeling approaches that target the mechanisms that generate behavior. The goal of modeling behavior is to construct the problem space and the design constraints within which a given range of behaviors can be viewed. This will in turn provide hypotheses about the neural implementation of the mechanisms.

## Behavior, what else?

Remarkably, what seems to be missing most in the current landscape is a focus on the study of behavior itself. It is interesting that neuroscientists and the brain imaging community have recently stressed the need for a renewed focus on behavior (Krakauer et al., 2017). For emotion researchers wary of the limited explanatory value of many current brain imaging studies, what is most lacking is a clear link to the behavior presumably related to the observed brain activation. When one looks at what emotion scientists have been doing for the last fifty years, especially the last two decades in which we have witnessed enormous leaps in available technologies, there has been very little behavioral research. The design imperatives of fMRI experiments often require downgrading our designs compared to the level of sophistication known from the classical psychophysics experiments and this in turn offers very few constraints on models of neural implementation. There is precious little work so far that consists of systematic behavior observation and model based manipulation of hypothetical variables. Somehow a balance will need to be struck between importing mental modules from cognitive psychology of the eighties into the scanner and rejecting the need for theory in favor of radical empiricism of neural network learning models. The following are some of the important stepping-stones.

### Going forward with the study of natural behavior

Emotional behavior takes place in the natural habitat of the organism. Behavior that is typically qualified with one or another emotion label, affective quality etc., is, like all behavior phylogenetically and ontogenetically shaped. A substantial degree of phylogenetic adaptiveness with respect to the habitat is part and parcel of the description of behavior whenever emotional predicates are used. It would seem logical then to make that functional relation between organism, behavior and environment a core part of the analysis of emotion. A striking fact is that we currently have very little understanding of how humans behave in their natural environments. Animal studies have an edge here because, as researchers cannot avail themselves of shortcuts based on the use of language, hypotheses and explanations have to be cast as descriptions of behavior. In human studies we have traditionally relied on language.

At present we have barely made a beginning with the empirical study of the emotional life of organisms in their natural (including social) environment. We need the kind of experiments that the first generation of ethologists were involved in, unpacking the specific properties of the behavior, the conditions under which it occurs etc., with a degree of attention to detail that is still largely absent in the tradition of human emotion research.

There are as of yet very few examples of this kind of work. For example, recent animal experiments (Gross and Canteras, 2012) and human fMRI studies (Mobbs and Kim, 2015) have introduced a powerful new dimension to the familiar animal or human fear experiments by looking at the role of distance between the subject and the fear triggering object and in doing so they revealed that different brain systems are entering into play as a function of this distance. We have followed a similar logic by using VR technology in the scanner (de Borst et al., under review). This indicates that fear and fear behavior are abstractions that have to be unpacked into different component mechanisms. Each is then to be understood in its functional role given the context for which they are designed, rather than used as landmarks for locating neural correlates of emotion concepts.

### Study the production of behavior rather than passive perception

In a sense emotion scientists have put the cart before the horse by almost exclusively studying perception rather than production. Yet it is difficult to study perception in the absence of a model of production that would give some guidance on the parameters. There currently are a few descriptive models of whole body movements based on visual categories of movements and some using muscle measurements. For example, the Facial Action Coding System (FACS) describes a wide range of muscles involved in the production of facial expressions yet this descriptive tool has not really guided detailed naturalistic research. Instead of using this measuring method as a tool for discovery, the facial muscles were clustered in a set of basic emotions and the assumption is that these are the templates that drive recognition. Instead, equipped with an extensive description of the facial and bodily musculature and without the filter of basic emotion theory, a new breed of emotion ethologist and anthropologist must go out into the wild and develop novel ways to study the relationship of multiple facial muscle patterns with actual behavior contexts.

### Include other modalities besides vision

Facial expressions remain the stimuli of choice in emotion research (de Gelder, 2016). Some studies have turned to scenes, bodies, voices, or touch but they were too often motivated by the notion of an abstract a-modal conceptual representation. But rather than setting up the experiments with the goal of finding what is common to the different channels of emotion communication, one should focus on the differences related to the specific behavioral functionality. For example, one should expect that affective signals by the face, voice or whole body operate differently, depending on, for example, the distance from the perceiver.

### Beyond the traditional scope of emotion studies

Emotion theorists traditionally address conventional emotional triggering situations that allow for easy presentation using computer screens in the lab. This is rather unnatural but even more important, the range of emotion is very restricted. The scope of emotion research must be widened to include affective experiences in the areas of the arts, of cultural, political and religious practices, all of which are potent triggers of affective behavior.

### Integrate modeling approaches and experiments

Among computational neuroscientists there is increasing interest in modeling brain processes. But obviously this cannot simply be a matter of replacing psychological with computational models. What we need now is proposals for modeling behavior that will elaborate on the link between descriptions of behavior and the face where we have descriptions at the muscle level, visual studies, developmental and patient studies.

### Endorse radically novel methodologies

Virtual Reality (VR) is an example of a powerful new technology that has seduced game makers but not yet scientists, with few recent exceptions in the area of navigation. VR Specialists have long underscored how the feeling of presence can be created with these tools. Current VR tools must be adapted for use in basic scientific research. They promise complete control over the sensory environment and the possibility to manipulate selectively the experimental variables and measure the behavioral correlates. In addition to the opportunity to study behavior in a quasi-natural environment, there are significant methodological and theoretical advantages to using realistic VR. At the core of the VR experience is not only the sensation of the present experience (e.g., “I scream because I am afraid of that bear”) but at the same time, another less noted aspect, the suspension of belief or the cognition bypass created by VR. When entering a VR environment, the participants know that what they experience is not real, yet they display “automatically” intense and context-appropriate emotions. The experience of presence goes hand in hand with a suspension of belief yet the emotions are experienced for “real,” when subjectively reported as well as when assessed with physiological and brain imaging methods. This makes VR a good method for investigating the behavioral repertoire of an organism.

## Some corollaries

How does the current proposal relate to the classical debates we mentioned at the beginning? The very idea of limiting emotion science to descriptions of behavior triggers classical fears of reductionism. Behavioral descriptions are seen as ignoring the need for and the complexity of mental state attributions. But descriptions of behavior need not result in the reductionist or revisionist position along the old behaviorism/mentalism divide. We need to develop an ethological, anthropological approach to the study of human emotion.

### Beyond basic emotions and their neural basis

From a design perspective the debate on whether or not basic emotions exist is not especially relevant any more. The concepts from traditional emotion theory can continue to be useful as first indications in a field of study but they are neither descriptive nor explanatory. Fear can very well continue to be a good example of the kind of research that adheres to functional design principles, and inspire the search for them. But that research is not to be confused with or mistaken as providing answers for the subjective comprehension. It would be a mistake to ascribe to organisms comprehension of the competence that is being described by the theory. Post-behaviorism created a climate for blueprints on how cognition and emotion were organized in the functional architecture of the brain. Inspired by cognitive models and functional dissociations observed in brain damaged patients, cognitive neuroscientist expected that memory processes would eventually be localized to memory circuits, sensory processes to sensory circuits, motor processes to motor circuits, and emotion processes would eventually be localized to emotion areas. Interestingly, this research program did not boost progress in studying the variety and complexity of affective behavior and the data basis of human emotion research remains still very thin.

### No privileged access

Introspection, the fountain of all the splendors and miseries of emotion research, is not a shortcut to get at processes, a window on the feelings or even a tool for constructing emotions. Introspection itself is behavior and should be investigated as such with empirical methods. This appears difficult to accept, specifically in the area of emotions. Many researchers keep to the traditional picture that we have feelings and thoughts of which we are conscious and view this link with subjective experience and consciousness of it as constitutive for emotions. This makes introspection (and interoception) a crucial component of any emotion theory that aims to go beyond a description of organismic processes. It is this assumption that perpetuates the crippling dualism that is at the core of a lot of current confusion.

Instead, we must see introspection for what it is, a behavior with its own biological roots, designed for specific purposes and behavioral competence that is to be studied and explained like any other behavior. Ultimately, if we want to transform what introspective and interoceptive reports deliver into scientific cash, we must step back and view it introspective behavior and subjective emotion building experience also from the standpoint of design-without-a-designer.

### Facts vs. norms

In arguing that introspection is itself a kind of behavior and should be studied as such, from a third person perspective, rather than by taking first person authority for granted, we have so far ignored its normative side. Introspection as a practice we know in daily life is at the service of explaining and justifying our actions to ourselves in the course of constructing ourselves as moral agents. I submit that this practical in the sense of practice-based facet of introspection, is traditionally the domain of moral philosophy and of ethics, We cannot build upon it a methodology in emotion science. Some questions are mistakenly viewed as falling within the scope emotion sciences while in fact they touch upon these practical or practice-based issues. To phrase it in Kantian terms, these are issues that pertain to the question “What must I do,” rather than to the question “What can I know.” A persistent confusion between these two is the source of much confusion about the goals of emotion theory. Answers to the latter question surely *inform* answers to the first but do not *dictate* them.

## Conclusion

Researchers outside emotion science still tend to hold emotion scientists to a definition of what emotions are, as if an answer to that question would guarantee the homogeneity and specificity of emotion science. But a maturing emotion science cannot cling to the notion that there is or that there should be a single definition or a single conceptual core to all fields of emotion science. As scientists, we know that the world is bigger than our concepts. In future developments one should not take for granted that the different specialties and application domains under the very wide and leaky umbrella of emotion science, have or must have much in common because presumably they are all about basic emotions. To be true to this, we need to be on the lookout for new questions, not for answers to the old ones. Emotion science is a loosely organized collection of research endeavors each working with its own notion of emotion. Just how all these hang together is a long-term empirical question, not simply a matter of the definition of basic emotions, nor of the denial of their existence. Similar to what is the accepted situation in physics, it is unlikely that all processes and states we commonly associate with emotions will come together in a unified science of emotion.

It is early days for emotion science. The dichotomies mentioned above are not the horns of a dilemma that emotion scientists are forever stuck with. Current debates seem to force a choice between mechanistic explanations of the organism, combined or not with taking up residence in the realm of subjective experience formatted by language, social processes and culture. But arguing that emotions are social or cognitive constructions only makes scientific sense when studying such constructions themselves as behavior, and thereby subject them the same type of functional designer approach advocated here. In other words, we need to apply the same approach to the scientific study of subjective, interoceptive and introspective behavior as is applied to the study of any other behavior of the organism. That is, methodologies of introspection (e.g., qualitative methods, questionnaires, implicit association tests and the like) using self-report and self-observation are not simply sources of data but are themselves behaviors. Self-understanding, whether viewed as rooted in subjective mindreading, in interoceptive signal reading or in constructive conceptual acts cannot be where the bucket stops because those activities, whether directed at one's own mind or at others, are also behavior. As such they are not at the heart of the solution to the body-mind problem but part of it and to be subjected to the kind of design-based explanation sketched here.

This journal encourages submission of experimental and theoretical studies that report new research in all of the above areas. We welcome contributions that challenge the current status quo in the field of emotion research and look forward to explore novel phenomena in innovative ways.

## Author contributions

The author confirms being the sole contributor of this work and approved it for publication.

### Conflict of interest statement

The author declares that the research was conducted in the absence of any commercial or financial relationships that could be construed as a potential conflict of interest.

## References

[B1] Adolphs R. (2016). How should neuroscience study emotions? By distinguishing emotion states, concepts, and experiences. Soc. Cogn. Affect. Neurosci. 12, 24–31. 10.1093/scan/nsw153 27798256 PMC5390692

[B2] Barrett L. F. (2017). Functionalism cannot save the classical view of emotion (short version). Soc. Cogn. Affect. Neurosci. 12, 34–36. 10.1093/scan/nsw156 27798259 PMC5390699

[B3] Barrett L. F. Lewis M. Haviland-Jones J. M. (2016). Handbook of Emotions. New York, NY: Guilford Publications.

[B4] Carruthers P. (2016). Consciousness, higher-order theories of, in The Stanford Encyclopedia of Philosophy (2001/2007/2011), ed Zalta E. (Stanford, CA: CSLI).

[B5] Damasio A. (1999). The Feeling of What Happens: Body and Emotion in the Making of Consciousness. New York, NY: Harcourt Brace.

[B6] de Gelder B. (2016). Emotions and the Body. New York, NY: Oxford University Press.

[B7] Dennett D. C. (2017). From Bacteria to Bach and Back: The Evolution of Minds. New York, NY: W. W. Norton & Company.

[B8] Fodor J. (1968). Psychological Explanation. New York, NY: Random House.

[B9] Griffits P. E. (1997). What Emotions Really Are. Chicago, IL: University of Chicago Press.

[B10] Gross C. T. Canteras N. S. (2012). The many paths to fear. Nat. Rev. Neurosci. 13, 651–658. 10.1038/nrn3301 22850830

[B11] Krakauer J. W. Ghazanfar A. A. Gomez-Marin A. MacIver M. A. Poeppel D. (2017). Neuroscience needs behavior: correcting a reductionist bias. Neuron 93, 480–490. 10.1016/j.neuron.2016.12.041 28182904

[B12] LeDoux J. (2015). Anxious: Using the Brain to Understand and Treat Fear and Anxiety. New York, NY: Penguin.

[B13] Mobbs D. Kim J. (2015). Neuroethological studies of fear and risky decision-making in rat and humans. Curr. Opin. Behav. Sci. 5, 8–15. 10.1016/j.cobeha.2015.06.005 PMC6034691 29984261

